# Clinical features of stroke mimics in the emergency department

**DOI:** 10.1002/ams2.338

**Published:** 2018-04-10

**Authors:** Yuichi Okano, Kazuaki Ishimatsu, Yoichi Kato, Junichi Yamaga, Ken Kuwahara, Katsuki Okumoto, Kuniyasu Wada

**Affiliations:** ^1^ Department of Emergency Medicine Kumamoto Red Cross Hospital Kumamoto Japan

**Keywords:** National Institutes of Health Stroke Scale, stroke, tissue‐type plasminogen activator

## Abstract

**Aim:**

To clarify the features of stroke mimics.

**Methods:**

We retrospectively investigated stroke mimic cases among the suspected stroke cases examined at our emergency department, over the past 9 years, during the tissue‐type plasminogen activator treatment time window.

**Results:**

Of 1,557 suspected acute stroke cases examined at the emergency department, 137 (8.8%) were stroke mimics. The most common causes were symptomatic epilepsy (28 cases, 20.4%), neuropathy‐like symptoms (21 cases, 15.3%), and hypoglycemia (15 cases, 10.9%). Outcomes were survival to hospital discharge for 91.2% and death for 8.8% of the cases. Clinical results were significantly different between stroke mimics and the stroke group for low systolic blood pressure, low National Institutes of Health Stroke Scale score on initial treatment, history of diabetes, and no history of arrhythmia. On multivariate analysis, distinguishing factors for stroke mimics include systolic blood pressure ≤ 140 mmHg, National Institutes of Health Stroke Scale score ≤ 5 points, history of diabetes, and no history of arrhythmia.

**Conclusions:**

Frequency of stroke mimics in cases of acute stroke suspected cases is 8.8%, and the most common cause is epilepsy. In order to distinguish stroke mimics, it is useful to understand common diseases presenting as stroke mimics and evaluate clinical features different from stroke by medical interview or nerve examination.

## Introduction

Stroke is a condition that most frequently requires nursing care in Japan.^1^ Recently, the functional prognosis of patients with cerebral infarction is improved by treatment with tissue‐type plasminogen activator (t‐PA)^2^ and thrombus retrieval^3^ by intracerebral intravascular therapy for acute stage cerebral infarction.^4^ Therefore, the emergency department (ED) is required to provide a new system capable of rapid diagnosis and treatment decisions for acute stage cerebral infarction.

At the Kumamoto Red Cross Hospital (Kumamoto, Japan), in accordance with the “rt‐PA (alteplase) Intravenous Therapy Appropriate Treatment Guidelines”^5^ announced by the Japan Stroke Society, emergency physicians and neurologists have worked together to create a special system called the “t‐PA mode” to carry out acute stroke care. The t‐PA mode is issued by the emergency physicians at the time of entering information regarding patients carrying the possibility of t‐PA treatment indications, and emergency physicians prioritize neurological examination and imaging (whole cervical ultrasound, computed tomography, and magnetic resonance imaging [MRI] enforced), to obtain an interval of 1 h from ambulance delivery to t‐PA. The neurologists then decide whether t‐PA is indicated based on these imaging results and clinical symptoms. The t‐PA mode system has been implemented for rapid stroke treatment in our hospital since 2006. However, “stroke mimics” may present among patients suspected with acute stroke in the ED.

Stroke mimics is a term indicating a pathological condition that shows a stroke‐like clinical picture due to a symptom caused by a disease other than cerebrovascular diseases.^6^ The causes of stroke mimics are diverse and are mainly metabolic, such as hypoglycemia, hepatic encephalopathy, and neurological diseases, such as encephalitis and brain tumor.^7^ Treatment with t‐PA for stroke mimics is not indicated; in particular, acute aortic dissection and spinal cord epidural hematoma are contraindicated because these diseases worsen with t‐PA treatment.^8^ Therefore, distinguishing stroke mimics from stroke is important to avoid unnecessary acute treatment and secondary prevention at the ED. However, few studies have reported useful clinical findings for distinguishing stroke mimics from stroke, and the method of discrimination has not yet been clarified. Therefore, we investigated cases of stroke mimics experienced at our center and clarified the clinical features of stroke mimics and the factors to differentiate between stroke mimics and stroke.

## Methods

### Patients and study design

We carried out a single‐center retrospective cohort study of patients treated in the ED at Kumamoto Red Cross Hospital (Kumamoto, Japan) from April 2006 to March 2015. We included patients (1,691 cases) admitted in the ED who were suspected with acute‐stage stroke (within the time that the onset was indicated for t‐PA treatment, so‐called “t‐PA mode” cases).

According to the t‐PA mode manual in our hospital, these enrolled subjects underwent blood, cervical ultrasound, computed tomography, and MRI examinations (all examinations were carried out if not contraindicated). We excluded patients whose onset of symptoms and diagnosis were unknown or who were found to have contraindications for t‐PA treatment after ED assessment. Finally, we examined 137 patients who had a final diagnosis of stroke mimic.

**Figure 1:**
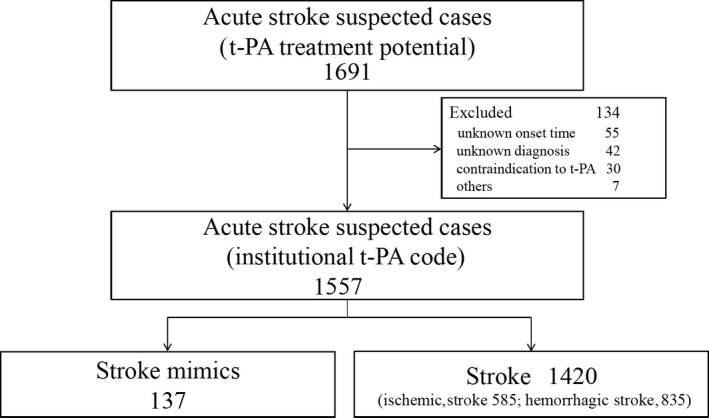
Background of this single‐center retrospective cohort study of patients treated in the emergency department with suspected acute‐stage stroke. t‐PA, tissue‐type plasminogen activator.

In addition, we classified the enrolled patients into the stroke mimics group (137 patients) and stroke group (1,420 patients, consisting of 835 patients [59%] with hemorrhagic stroke and 585 patients [41%] with ischemic stroke), and the two groups were compared (Fig. 1).

The study was undertaken in accordance with the Declaration of Helsinki, and the protocol was approved by the Institutional Review Board of Kumamoto Red Cross Hospital.

### Methods

We carried out a retrospective study using data from emergency medical services (EMS) records, medical records at our hospital, summary during hospitalization, and the t‐PA mode ledger. Based on the predictors of stroke mimics reported by Merino *et al*.,^8^ the following data were collected: age, sex, vital signs, National Institutes of Health Stroke Scale (NIHSS) score, transport means, arrival at ED, medical history with vascular risk (hypertension, diabetes, hyperlipidemia, arrhythmia, and smoking), causes of stroke mimics, and outcome.

All measurements were carried out at the time of initial treatment at our hospital. The NIHSS assessment was adopted by neurologists alone, or was checked by neurologists, to ensure the quality of neurological examination. The medical history was obtained in the medical record or hospital summary. The period and presence or absence of treatment are not mentioned in this study.

### Statistical analyses

We undertook statistical analyses using IBM spss Statistics 22 (IBM, Armonk, New York, USA). We described the characteristics of stroke mimics from survey data, and extracted factors related to stroke mimics and stroke differentiation with logistic regression analysis.

For the test method, the values between the two groups were compared using the Mann–Whitney *U*‐test for real data, and the χ^2^‐test for categorical data. A *P*‐value <0.05 was considered statistically significant.

## Results

### Background

The median age of the patients was 69.1 ± 27.4 years, and most of the patients were men (85 men and 52 women). The risk factors for stroke were as follows: hypertension in 55 (40.1%) cases, smoking in 39 (28.5%), diabetes in 28 (20.4%), hyperlipidemia in 17 (12.4%), and arrhythmia in 17 (12.4%) (Table 1).

**Table 1:** Baseline characteristics, clinical history, and cause of stroke mimic in patients arriving at the emergency department (ED) with suspected stroke

Variable	Stroke mimics group (*n* = 137)
Sex, male : female (% male)	85:52 (62.0%)
Age, mean ± SD	69.1 ± 27.4
Vital signs	
Systolic blood pressure ± SD, mmHg	143 ± 36
Diastolic blood pressure ± SD, mmHg	80 ± 23
Japan Coma Scale (JCS), *n* (%)	
JCS 0	51 (37.2)
JCS 1–3	60 (43.7)
JCS 10–30	12 (8.8)
JCS 100–300	14 (10.2)
NIHSS, mean ± SD	7.3 ± 8.9
Vomiting	23 (16.8)
Transportation means, *n* (%)	
Ambulance	106 (77.4)
Helicopter emergency medical service	7 (5.1)
Walk‐in	24 (17.5)
Arrival at ED, *n* (%)	
00:00–07:59	23 (16.8)
08:00–15:59	68 (49.6)
16:00–23:59	46 (33.6)
Medical history, *n* (%)	
Hypertension	55 (40.1)
Diabetes mellitus	28 (20.4)
Hyperlipidemia	17 (12.4)
Arrhythmia	17 (12.4)
Tobacco use	39 (28.5)
Cause of stroke mimic, *n* (%)	
Epileptic seizure	28 (20.4)
Psychiatric diagnosis	21 (15.3)
Hypoglycemia	15 (10.9)
Acute aortic dissection	13 (9.5)
Syncope	9 (6.6)
Sepsis	9 (6.6)
Drug intoxication	8 (5.8)
Brain tumor	7 (5.1)
Acute alcohol intoxication	5 (3.6)
Cervical spondylosis	5 (3.6)
Arteriosclerosis obliterans	3 (2.2)
Hepatic encephalopathy	3 (2.2)
Peripheral neuropathy	2 (1.5)
Cervical epidural hematoma	2 (1.5)
Behçet's disease	1 (0.7)
Systemic lupus erythematosus	1 (0.7)
Multiple sclerosis	1 (0.7)
Multifocal neuropathy	1 (0.7)
Hyperosmolar hyperglycemic non‐ketotic coma	1 (0.7)
Heat stroke	1 (0.7)
Lumbar compression fracture	1 (0.7)

NIHSS, National Institutes of Health Stroke Scale; SD, standard deviation.

### Baseline characteristics and clinical history

As detailed in Table 1, the patients were transported by ambulance, helicopter EMS (HEMS), and direct emergency outpatient (walk‐in) visits in 106 (77.4%), 7 (5.1%), and 24 (17.5%) cases, respectively. The level of consciousness based on the Japan Coma Scale during the initial medical examination was I (0,1,2,3), II (10,20,30), and III (100,200,300) in 51 (37.2%), 60 (43.7%), 12 (8.8%), and 14 (10.2%) patients, respectively. The mean (± standard deviation) NIHSS score during the initial medical examination was 7.3 ± 8.9. The systolic and diastolic blood pressure was 143 ± 36 mmHg and 80 ± 23 mmHg, respectively. In addition, 23 (16.8%) patients were vomiting during the initial medical examination.

### Cause of stroke mimics

The most frequent causes of stroke mimics were symptomatic epilepsy (28 cases, 20.4%), psychiatric disorders, such as hysteria and anxiety neurosis (21 cases, 15.3%), hypoglycemia (15 cases, 10.9%), and acute aortic dissection (13 cases, 9.5%). Other causes of stroke mimics included various diseases, such as infectious diseases, drug addiction, and alcohol‐related diseases (Table 1).

### Outcomes

**Figure 2:**
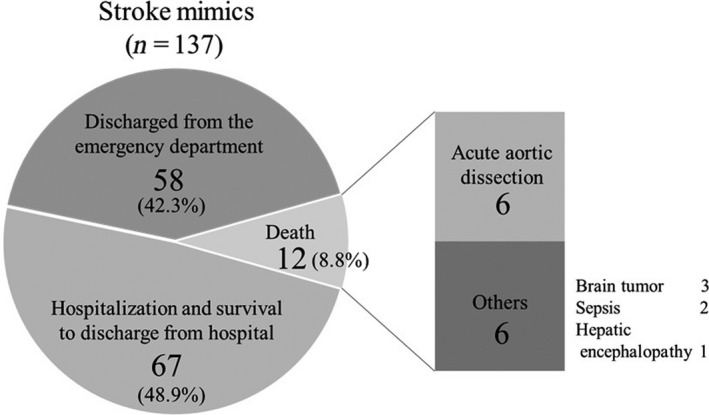
Outcomes of 137 patients admitted to the emergency department with stroke mimics.

In terms of the outcomes of stroke mimics, the symptoms of 58 (42.3%) patients were relieved, and the patients were discharged on the same day, whereas 67 (48.9%) patients were hospitalized on the same day and were discharged or transferred to another hospital (Fig. 2). Twelve (8.8%) patients died.

The most common cause of death was acute aortic dissection (6 of 13 patients, 46.1%), half of whom died suddenly in the ED. Other causes of death were brain tumor (three patients), sepsis (two patients), and hepatic encephalopathy (one patient).

### Analysis of factors associated with stroke mimics

Univariate analysis showed that the five factors showing a significant difference between the stroke mimics and stroke groups were systolic blood pressure (143 ± 36 mmHg versus 172 ± 34 mmHg; *P* < 0.001), diastolic blood pressure (80 ± 23 mmHg versus 94 ± 21 mmHg; *P* = 0.002), NIHSS score during the initial medical examination (7.3 ± 8.9 versus 11.9 ± 8.8; *P* = 0.015), history of diabetes (20.4% versus 13.1%; *P* = 0.014), and absence of arrhythmia (87.6% versus 78.0%; *P* = 0.007; Table 2).

**Table 2:** Univariate analysis of factors associated with stroke mimics

Variable	Stroke mimics group (*n* = 137)	Stroke group (*n* = 1,420)	OR	95% CI	*P*‐value
Sex, male : female (% male)	85: 52 (62.0%)	859: 561 (60.4%)	1.1	0.74–1.53	0.71
Age, mean ± SD	69.1 ± 27.4	70.8 ± 13.4			0.355
Vital signs					
Systolic blood pressure ± SD, mmHg	143 ± 36	172 ± 34			<0.001
Diastolic blood pressure ± SD, mmHg	80 ± 23	94 ± 21			0.002
Japan Coma Scale (JCS), *n* (%)					
JCS 0	51 (37.2)	501 (35.3)	0.9	0.67–1.39	0.85
JCS 1–3	60 (43.7)	558 (39.3)	1.2	0.84–1.71	0.294
JCS 10–30	12 (8.8)	236 (16.6)	0.6	0.31–1.07	0.081
JCS 100–300	14 (10.2)	125 (8.8)	1.2	0.65–2.08	0.602
NIHSS, mean ± SD	7.3 ± 8.9	11.9 ± 8.8			0.015
Vomiting, *n* (%)	23 (16.8)	206 (14.5)	1.1	0.68–1.75	0.7
Transportation means, *n* (%)					
Ambulance	106 (77.4)	1,128 (79.4)	1.3	0.81–2.12	0.26
Helicopter emergency medical service	7 (5.1)	80 (5.6)	0.9	0.41–1.99	0.793
Walk‐in	24 (17.5)	212 (14.9)	1.2	0.76–1.92	0.402
Arrival at ED, *n* (%)					
00:00–07:59	23 (16.8)	210 (14.8)	1.2	0.73–1.86	0.52
08:00–15:59	68 (49.6)	708 (49.9)	1	0.72–1.45	0.901
16:00–23:59	46 (33.6)	502 (35.4)	0.9	0.59–1.24	0.374
Medical history, *n* (%)					
Hypertension	55 (40.1)	682 (48.0)	0.7	0.51–1.04	0.08
Diabetes mellitus	28 (20.4)	186 (13.1)	1.7	1.09–2.65	0.014
Hyperlipidemia	17 (12.4)	223 (15.7)	0.8	0.45–1.29	0.29
Arrhythmia	17 (12.4)	312 (22.0)	0.5	0.30–0.85	0.007
Tobacco use	39 (28.5)	389 (27.4)	1.2	0.79–1.74	0.411

CI, confidence interval; NIHSS, National Institutes of Health Stroke Scale; OR, odds ratio; SD, standard deviation.

Subsequently, to adjust the influence of other variables, multiple logistic analyses were carried out by inputting factors that were significant, such as age and sex, and univariate analysis was carried out for independent variables. As a result, significant differences were found in four factors: (i) systolic blood pressure ≤140 mmHg; (ii) NIHSS score ≤5 points; (iii) history of diabetes mellitus; (iv) no history of arrhythmia. The clinical features of these four factors were suggested to be useful as factors related to stroke mimics and stroke differentiation (Table 3).

**Table 3:** Factors associated with differentiation of stroke mimics

Factor	OR	95% CI	*P*‐value
SBP < 140 mmHg	4.3	2.930–6.255	<0.001
NIHSS < 5	3.2	2.173–4.617	<0.001
No arrhythmia (medical history)	1.8	1.061–3.103	0.030
DM (medical history)	1.6	1.022–2.607	0.040

CI, confidence interval; DM, diabetes mellitus; NIHSS, National Institutes of Health Stroke Scale; OR, odds ratio; SBP, systolic blood pressure.

## Discussion

Our results suggest that 8.8% of stroke mimics occur among patients with suspected acute‐stage stroke who were admitted to the ED, and the frequent causes of stroke mimics were symptomatic epilepsy (28 cases, 20.4%), psychiatric disorders (21 cases, 15.3%), and hypoglycemia (15 cases, 10.9%). The frequency of stroke mimics varies greatly depending on the evaluator and evaluation time. The reported frequency of stroke mimics ranges widely, from 1.2 to 31%, in Europe;^9^ in Japan, the frequency is 6.7%, even if neurosurgical specialists are involved in patient examination.^10^ So it was suggested that stroke mimics is not uncommon diseases.

Furthermore, approximately half of the cases of stroke mimics in this study were accounted for by epilepsy, psychiatric disorders, and hypoglycemia. Because these are considered to be common diseases that could present as stroke mimics, we suggest that familiarizing with the clinical features of these diseases is necessary to differentiate it from stroke.

Zinkstok *et al*.^11^ reported that epileptic seizures (41%) and psychiatric disorders (28%) are the most common diseases that can cause stroke mimics, and the frequency of hypoglycemia is low (1%). Compared with our study, these reports showed a difference in the ratio of hypoglycemia, which might be because blood glucose measurement by EMS personnel was not widely used during the research period. As a result, the proportion of hypoglycemia, as a cause of stroke mimics, was increased in our study.

Moreover, the reason why symptomatic epilepsy was the most common cause of stroke mimics is that, after a seizure attack, Todd's paralysis occurs on one side, and its symptoms persist for several hours; these symptoms were considered to be misidentified as stroke symptoms. In this study, among 28 patients with symptomatic epilepsy, 13 (46.4%) patients were confirmed to have Todd's paralysis in the ED, and the duration of hemiplegic symptoms was 1.7 ± 1.3 h. Therefore, we speculate that many patients with symptomatic epilepsy with hemiplegia remained in the ED.

Furthermore, non‐convulsive status epilepticus, which is a pathological condition that suddenly results in speech disorders, cognitive impairment, and sensory disturbance without convulsions, is known as a type of epilepsy.^13^ Therefore, we considered that differentiating between non‐convulsive status epilepticus and stroke is more difficult.

In this study, we examined whether a difference in frequency of stroke mimics was found depending on the emergency transportation method, but no significant difference was observed between patients transported by ambulance and HEMS (8.6%) and walk‐in reception (10.2%) (*P* = 0.41). From this result, we considered that differentiating stroke mimics from stroke is difficult, even if prehospital care is carried out by the EMS and HEMS staff.

Subsequently, we discussed factors that differentiate stroke mimics from stroke. In this study, these determining factors were: (i) systolic blood pressure ≤140 mmHg; (ii) NIHSS score ≤5 points; (iii) history of diabetes mellitus; (iv) no history of arrhythmia. Several studies have reported on the predictors of stroke mimics (Table 4).^10^, ^14^, ^15^, ^16^, ^17^ Most reported that stroke mimics were characterized by low vascular risk factors in young individuals and low NIHSS scores.

**Table 4:** Clinical factors that predict the presence of stroke mimics

Registry (year)	City, state (country)	Period, years	Stroke, *n*	Stroke mimics, *n* (%)	Clinical factor
Libman *et al*. (1995)^14^	New Hyde Park, NY (USA)	1990–1992	333	78 (19)	Decreased consciousness No history of angina
Hand *et al*. (2006)^10^	Victoria (Australia)	2001–2004	241	109 (31)	No (not) cognitive impairment Abnormal signs in other systems suggested a mimic Exact time of onset Definite focal symptoms Abnormal vascular findings Presence of neurological signs Being able to lateralize the signs to the left or right side of the brain Being able to determine a clinical stroke subclassification
Winkler *et al*. (2008)^15^	Basel (Switzerland)	1998–2007	243	7 (2.8)	Seizure Lower NIHSS
Tsivgoulis *et al*. (2011)^16^	Phoenix, AZ (USA)	2003–2008	483	56 (10)	Younger age Lower median NIHSS
Chang *et al*. (2012)^17^	Los Angeles, CA (USA)	2007–2008	163	30 (15)	Focal weakness Computed tomographic angiography findings Precordialgia

NIHSS, National Institutes of Health Stroke Scale.

Compared with the predictors in this study, the NIHSS score was consistently low. In this study, the cause of the low NIHSS score in the stroke mimics group was that few patients had a severe consciousness disorder; the symptoms of hemiplegia were mild, wherein neurological symptoms (symptomatic epilepsy, syncope etc.) improved during the ED stay.

In addition, in this study, the cause of low systolic blood pressure among patients with stroke mimics was considered to be caused by diseases prone to hypotension, such as syncope, hypoglycemia, and sleeping medicine overdose. Gioia *et al*.^17^ reported that systolic blood pressure in a stroke group was higher than that in a stroke mimics group (146.1 mmHg versus 155.6 mmHg; *P* < 0.001) in the prehospital setting. This result was similar to our study, although the systolic blood pressure and conditions slightly differed immediately after entering the ED. Furthermore, Saver *et al*.^18^ reported that patients presenting with stroke mimics were characterized by younger age (64 ± 13 versus 70 ± 13, *P* = 0.06), low systolic blood pressure (141 mmHg versus 159 mmHg, *P* = 0.012), and mild neurological symptoms (NIHSS) (5.6 versus 9.3, *P* = 0.009). This result was also similar to the clinical symptoms of patients presenting with stroke mimics in our study. As previously mentioned, patients with stroke‐like symptoms, wherein the NIHSS score is relatively low and blood pressure is not elevated in the ED, should be treated carefully with the possibility of stroke mimics.

Subsequently, we examined the medical history of the stroke mimics group. Diabetes was found to be a discriminating factor because stroke mimics included numbness and dizziness of limbs due to prolonged effect of antidiabetic medications and diabetic neuropathy (e.g. peripheral nerve disorder and autonomic nervous disorder). Malouf *et al*.^19^ reported that approximately 9% of hypoglycemic patients present with neurological symptoms other than consciousness alteration. In this study, hypoglycemia was the third most common cause of stroke mimics. Therefore, undertaking hypoglycemic exclusion tests first for stroke‐like symptoms in the ED is important.

In contrast, the reason why no arrhythmia was mentioned as a differential factor is that atrial fibrillation in arrhythmia is an independent risk factor for ischemic stroke, and dizziness due to arrhythmia and cardiogenic syncope are less likely to be regarded as stroke‐like symptoms. Odutayo *et al*.^20^ reported that the risk of ischemic stroke was 2.33 times than that in people without atrial fibrillation in their cohort study. Of 955 patients with ischemic stroke in this study, 272 (28.5%) patients had atrial fibrillation. Because of the above findings, investigating the risk factors of stroke at an early stage, such as the presence of diabetes and arrhythmia (particularly atrial fibrillation), is necessary to evaluate stroke mimics.

As a method of discriminating stroke mimics from stroke, Goyal *et al*.^21^ proposed a scoring method called FABS. FABS included six variables: absence of facial droop, negative history of atrial fibrillation, age, systolic blood pressure at presentation, history of seizures, and isolated sensory deficit without limb weakness at presentation. They reported that a score of 3 points or more in the FABS system could identify patients with stroke mimics (sensitivity, 90%; specificity, 91%; Table 5). In addition, Ali *et al*.^22^ proposed a scoring method called Tele Stroke Mimic Score, with age, medical history (atrial fibrillation, hypertension, and convulsion), facial paralysis, and NIHSS score ≤14 as independent factors of stroke mimics. Because FABS and the Tele Stroke Mimic Score can all be evaluated with simple examination and vital signs, they may be evaluated in a short time even in a busy ED, and the necessity of carrying out head MRI can be judged from the scoring result.

**Table 5:** FABS scoring system^21^

Variable	Sensitivity, %	Specificity, %
Absence of facial droop	94	71
Age <50 years	53	86
Absence of atrial fibrillation	96	17
SBP <150 mmHg	73	74
Presence of isolated sensory deficit	15	97
History of seizure disorder	14	97

FABS ≥ 3 could identify patients with stroke mimics with 90% sensitivity (95% confidence interval, 86–93%) and 91% specificity (95% confidence interval, 88–93%).

SBP, systolic blood pressure.

Another method for discriminating stroke mimics from stroke is the copeptin (c‐terminal provasopressin) value, which is useful as a prognostic marker for patients with heart failure^23^ and head MRI diffusion‐emphasized images.^24^ Therefore, future research results could add blood test values and image findings as new discrimination factors for stroke mimics.

This study has several limitations. It was retrospective within only a single facility, the diseases that cause stroke mimics include diseases that have not reached definite diagnosis. Patients’ historical data were only obtained from the medical record, and the duration of the disease and treatment were not mentioned. In addition, because this study included cases of stroke mimics from the case data with t‐PA treatment indication, data of cases of stroke mimics wherein the onset of symptoms and final diagnosis were unclear could be missing.

In future studies, we should investigate how to better describe patients’ records in cases of stroke mimics, analyze the medical history in detail, and undertake multicenter collaborative research. As a result, new stroke mimics discrimination factors could be included, and it might be possible to create new scoring methods to differentiate stroke mimics.

## Conclusion

In this study, the frequency of stroke mimics in patients suspected with acute stroke was 8.8%, and the causes of stroke mimics were varied. Relevant factors that distinguish stroke mimics from stroke are: (i) systolic blood pressure ≤140 mmHg; (ii) NIHSS score ≤5; (iii) history of diabetes; (iv) no history of arrhythmia. These factors are suggested as useful findings in the diagnosis of stroke mimics in emergency medical care.

## Disclosure

Approval of the research protocol: The study's protocol was approved by the ethics committee of the Kumamoto Red Cross Hospital.

Informed consent: N/A.

Registry and the registration no. of the study/trial: N/A.

Animal studies: N/A.

Conflict of interest: None declared.
